# Supplementary material for: Common low complexity regions for SARS-CoV-2 and human proteomes as potential multidirectional risk factor in vaccine development
Source: BMC Bioinformatics. 2021 Apr 8;22:182. doi: 10.1186/s12859-021-04017-7 (PMC8027979; doi:10.1186/s12859-021-04017-7)
Supplement: Supplementary file 10 — Additional file 10. Table S10: Links to data and code generated or analyzed during this study. [file 12859_2021_4017_MOESM10_ESM.pdf]

Common low complexity regions for SARS-CoV-2 and human proteomes as potential multidirectional risk factor in vaccine development

Aleksandra Gruca[1], Joanna Ziemska-Legiecka[2], Patryk Jarnot[1], Elzbieta Sarnowska[3], Tomasz J. Sarnowski[2], Marcin Grynberg[2*]

[1]Department of Computer Network and Systems, Silesian University of Technology, Gliwice, Poland
[2] Institute of Biochemistry and Biophysics PAS, Warsaw, Poland
[3] Department of Molecular and Translational Oncology, Maria Sklodowska-Curie National Research Institute of Oncology, Warsaw, Poland

Corresponding author

E-mail: greenb@ibb.waw.pl (MG)

Key resource table

REAGENT or RESOURCE	SOURCE	IDENTIFIER
**Deposited data:**		
Replicase polyprotein 1a (pp1a)	COVID-19 UniProt resource	UniProt ID: P0DTC1
Replicase polyprotein 1ab (pp1ab)	COVID-19 UniProt resource	UniProt ID: P0DTD1
Spike glycoprotein (S)	COVID-19 UniProt resource	UniProt ID: P0DTC2
ORF3a protein (NS3a)	COVID-19 UniProt resource	UniProt ID: P0DTC3
Envelope small membrane protein (E)	COVID-19 UniProt resource	UniProt ID: P0DTC4
Membrane protein (M)	COVID-19 UniProt resource	UniProt ID: P0DTC5
ORF6 protein	COVID-19 UniProt resource	UniProt ID: P0DTC6
ORF7a protein	COVID-19 UniProt resource	UniProt ID: P0DTC7
ORF7b protein	COVID-19 UniProt resource	UniProt ID: P0DTD8
ORF8 protein	COVID-19 UniProt resource	UniProt ID: P0DTC8
Nucleoprotein (N)	COVID-19 UniProt resource	UniProt ID: P0DTC9
ORF9b protein	COVID-19 UniProt resource	UniProt ID: P0DTD2
ORF14 protein	COVID-19 UniProt resource	UniProt ID: P0DTD3
Hypothetical ORF10 protein	COVID-19 UniProt resource	UniProt ID: A0A663DJA2
**Software and algorithms**		
GBSC	https://github.com/pj504a/GBSC	N/A

MotifLCR	https://github.com/Addreoran/covid	(Ziemska-Legiecka 2019)
LCR-BLAST	https://drive.google.com/drive/folders/1lhQm5y5V0o82LXUVJN0qiGrwcEWUJp2e?usp=sharing	(Jarnot et al. 2020)
Wublast	http://blast.wustl.edu/	N/A
SEG	ftp://ftp.ncbi.nlm.nih.gov/blast/executables/blast+/LATEST	(Wootton and Federhen 1993)
blastp	ftp://ftp.ncbi.nlm.nih.gov/blast/executables/blast+/LATEST	(Altschul et al. 1990)
